# Metal-functionalized single-walled graphitic carbon nitride nanotubes: a first-principles study on magnetic property

**DOI:** 10.1186/1556-276X-6-97

**Published:** 2011-01-19

**Authors:** Hui Pan, Yong-Wei Zhang, Vivek B Shenoy, Huajian Gao

**Affiliations:** 1 Institute of High Performance Computing, 1 Fusionopolis Way, 138632, Singapore; 2 Division of Engineering, Brown University, 610 Barus & Holley, 182 Hope Street, Providence, RI 02912, USA

## Abstract

The magnetic properties of metal-functionalized graphitic carbon nitride nanotubes were investigated based on first-principles calculations. The graphitic carbon nitride nanotube can be either ferromagnetic or antiferromagnetic by functionalizing with different metal atoms. The W- and Ti-functionalized nanotubes are ferromagnetic, which are attributed to carrier-mediated interactions because of the coupling between the spin-polarized *d *and *p *electrons and the formation of the impurity bands close to the band edges. However, Cr-, Mn-, Co-, and Ni-functionalized nanotubes are antiferromagnetic because of the anti-alignment of the magnetic moments between neighboring metal atoms. The functionalized nanotubes may be used in spintronics and hydrogen storage.

## Introduction

Applications of spin-based devices are so far limited to information storage, and most of the spin-related materials and devices still rely primarily on the spontaneous ordering of spins in the form of different types of magnetic materials. This situation is expected to change with successful development of spin-based electronics, or spintronics, the new kind of electronics that seeks to exploit, in addition to the charge degree of freedom, the spin of the carriers [1]. The primary requirement for spintronics is to have a system that can generate a current of spin-polarized electrons. To enable a host of new device applications, it is necessary to develop materials which should have (a) high Curie temperature, (b) controllable carrier density and mobility, and (c) easily magnetic doping [2]. Ideally, a semiconductor can be made magnetic by including ions that have a net spin into a semiconductor [3]. The doped semiconductors are referred as dilute magnetic semiconductors (DMSs) because only a small amount of magnetic ions is required to make the semiconductor magnetic. In recent years, considerable efforts have been devoted to the study of DMS materials. The search for DMSs has been focused on cation substitution of semiconductors with transition metal (TM) elements, where the Curie temperature of the doped semiconductor can be below or above room temperature, depending on the host materials, carrier concentration, and doping elements [4-11]. However, the clustering of transition metal and the formation of secondary phases in DMSs are obstacles to their practical applications in spintronics [12]. Although the cation substitution with two different elements and anion substitution may overcome the clustering issue [7,13-15], the observation of ferromagnetism in undoped semiconductor nanoparticles suggested that doping-induced defects also contributed to the magnetic moment [16-19]. Therefore, for the practical application of DMSs, a crucial prerequisite is to overcome the clustering of TM in DMS and control the magnetic property, which may be solved by cation-anion codoping method [20].

**Figure 1:**
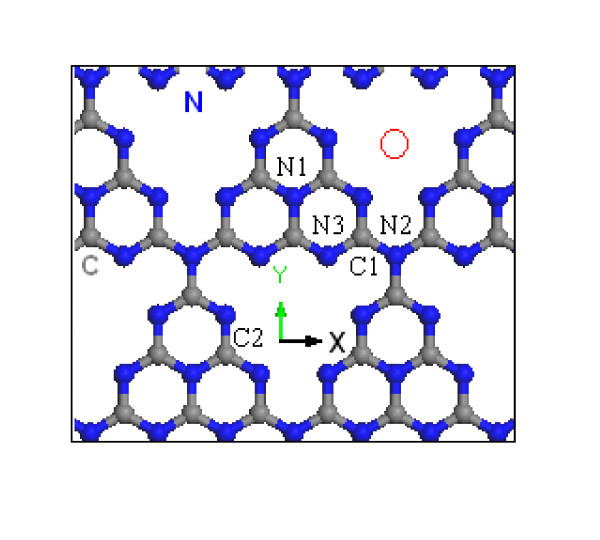
**(Color online) Atomic configuration of monolayer g-C_3_N_4_**. The wrapping vector of the nanotube is along the *x *axis. The red circle indicates the functionalizing site.

Carbon nitride has attracted considerable interest and has been widely used in electronic devices, thermoluminescence dosimeter, humidity sensor, coatings, and catalyst because of their interesting electronic, chemical, mechanical, and tribological properties [21-26]. Carbon nitride can exist in various phases not only depending on the C to N ratio, but also on atomic arrangements [21]. Among these phases, the graphitic carbon nitride (g-C_3_N_4_), a heptazine-based form, is regarded to be the most stable structure, and can be realized by thermo-condensation of C/N/H-containing precursors [22]. Recently, Gracia and Kroll [27] reported a new nanotube, g-C_3_N_4 _nanotube, based on density-functional-theory calculations. The unique porous structure of these materials (Figure 1) makes the TM doping controllable and the clustering avoidable because each pore can only host one atom. In this study, we present our first-principles study on the metal-functionalized g-C_3_N_4 _nanotubes. We show that the ferromagnetic g-C_3_N_4 _nanotube can be achieved by functionalizing the g-C_3_N_4 _nanotube with non-magnetic atoms.

## Methods

The first-principles calculation was carried out based on the density function theory (DFT) and the Perdew-Burke-Eznerhof generalized gradient approximation (PBE-GGA) [28,29]. The projector augmented wave (PAW) scheme as incorporated in the Vienna *ab initio *simulation package (VASP) was used [30,31]. The Monkhorst and Pack scheme of k-point sampling was used for integration over the first Brillouin zone [32]. The geometry of the g-C_3_N_4 _monolayer was first optimized to obtain the lattice constants with a vacuum space of 12 Å used to minimize the inter-layer interaction. A 5 × 1 × 1 grid for k-point sampling and an energy cutoff of 400 eV were used for the bulk and monolayer. The g-C_3_N_4 _nanotube is obtained by rolling up the g-C_3_N_4 _monolayer into a cylinder along the axial (*x*) direction (Figure 1), which is defined as a zigzag tube, adopting a similar terminology used in other nanotubes [33,34]. In our study, we chose a nanotube with an index of (4, 0), labeled as g-C_3_N_4_-zz4. A 1 × 1 × 3 grid for k-point sampling and an energy cutoff of 400 eV were consistently used in our calculations. Excellent convergence was obtained using these parameters, and the total energy was converged to 2.0 × 10^-5 ^eV/atom. A large supercell dimension with a wall-wall distance of 10 Å in the plane perpendicular to the tube axis was used to avoid any interaction between the nanotube and its images in neighboring cells.

The functionalization was achieved by positioning the metal elements at the center of triangular pores, one atom per pore, labeled as g-C_3_N_4_-zz4-TM (Figure 1). The TMs, including Ti, Cr, Mn, Fe, Co, Ni, and W (widely used doping elements in DMS), were used for the functionalization. The formation energy was estimated from

$$E_{\text{f}}=E_{\text{tot}}(\text{tube}+\text{doping})-E_{\text{tot}}(\text{tube})-n\mu_{\text{doping}}$$

where *E*_tot_(*tube *+ *doping*) and *E*_tot_(tube) are total energies of the g-C_3_N_4 _with and without doping, respectively; *μ*_doping _is the chemical potential of the functionalizing metal element, calculated from the metal bulk, and *n *is the number of the metal atoms.

## Results and discussion

**Figure 2:**
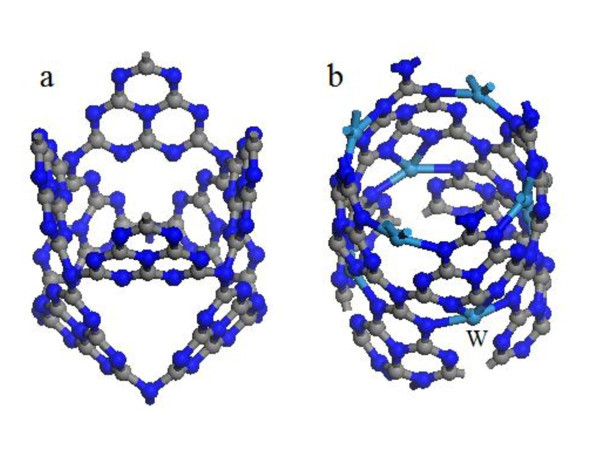
**(Color online) Geometries of (a) pure and (b) metal-functionalized g-C_3_N_4_-zz4 after optimization**.

After geometry optimization, the calculated in-plane repeating period of the g-C_3_N_4 _monolayer in the *x *direction is 7.129 Å, consistent with the experimental value [19]. For the nanotube, it is seen that the heptazine structures in the optimized geometry of g-C_3_N_4_-zz4 remain almost as flat as in the monolayer after the optimization (Figure 2a). A large curvature with the up C-N-C angle reduced to 106° is observed at the heptazine-heptazine N connection, consistent with the literature [21]. However, the lattice distortion both in the heptazine and at the connection is less than 0.5%. The optimized g-C_3_N_4_-zz4-TM (TM is Ti, Cr, Mn, Fe, Co, Ni, or W) (see Figure 2b) looks more like a cylinder than the unfunctionalized g-C_3_N_4_-zz4 (Figure 2a). The curvature in the g-C_3_N_4_-zz4-TM is uniform and the heptazine structure is not a plane. Interestingly, TM atom forms four bonds with the four neighboring edge nitrogen atoms with a bonding length of approximately 2.1 Å and a distance of about 2.7 Å from the other two edge nitrogen atoms.

The calculated functionalization energies are 0.14, 3.19, 0.57, 1.72, 2.69, 2.11 and 4.05 eV for Ti, Cr, Mn, Fe, Co, Ni, and W, respectively (see Table 1), indicating that the functionalization of Ti is relatively easy than that of other elements because of the lower formation energy. From the calculated exchange energies (the energy difference between anti-ferromagnetic and ferromagnetic states, *E*_exch _= *E*_AFM _- *E*_FM_) of the metal-functionalized g-C_3_N_4_-zz4 (see Table 1), we can see that W and Ti favor the ferromagnetic coupling with the exchange energies of 180 and 95 meV/pair of TMs, respectively. The Fe-functionalized nanotube should be non-magnetic due to the weak exchange energy (4 meV/pair of TMs). Cr-, Mn-, Co-, and Ni-functionalized nanotubes appear to be anti-ferromagnetic because the energies of anti-ferromagnetic state are lower than those of ferromagnetic state (see Table 1).

**Table 1:** Calculated functionalization energies, exchange energies, and magnetic moments of the metal-functionalized g-C_3_N_4 _nanotubes

	Ti	Cr	Mn	Fe	Co	Ni	W
*E*_f _(eV)	0.14	3.19	0.57	1.73	2.69	2.11	4.05
*E*_exch _(meV)/pair	95	-98	-7	4	-21	0	180
Moment (_μB_/M)	2.88	-	-	-	-	-	3.82

**Figure 3:**
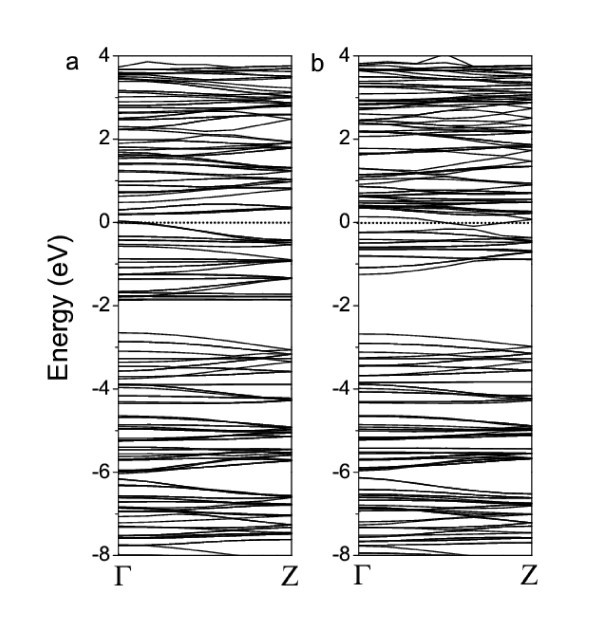
**Band structures of the W-functionalized g-C_3_N_4_-zz4**. **(a) **spin-up and **(b) **spin down. The Fermi level is at 0 eV.

**Figure 4:**
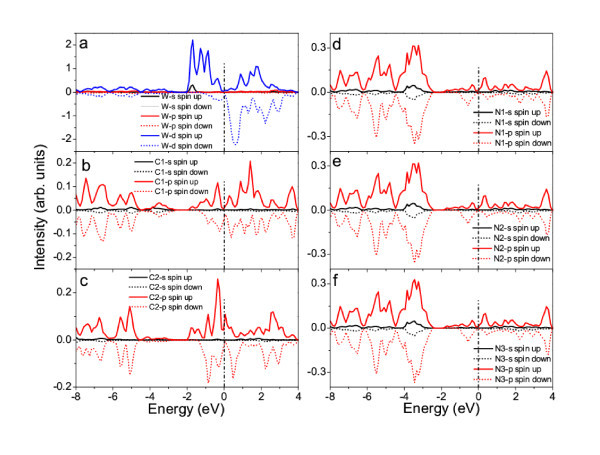
**Calculated partial density states (PDOS)**. W **(a)**, C **(b) **and **(c) **C, and N **(d)**, **(e) **and **(f) **in W-functionalized g-C_3_N_4_-zz4. The Fermi level is shifted to 0 eV (dot-dash line).

**Figure 5:**
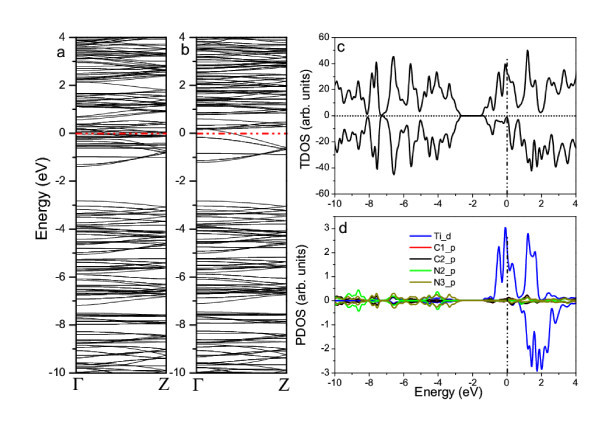
**Calculated electronic structures of the Ti-functionalized g-C_3_N_4_-zz4**. **(a) **spin-up and **(b) **spin-down band structures, **(c) **total density of states (TDOS), and **(d) **partial density of states (PDOS). The Fermi level is at 0 eV.

Figure 3 shows the calculated band structures of W-functionalized g-C_3_N_4_-zz4. The non-symmetrical electronic structures between spin-up and spin-down states demonstrated the ferromagnetism of g-C_3_N_4_-zz4-W. The band structures of both spin-up and spin-down states show heavily doped n-type semiconducting behavior with a direct band gap of 0.75 and 0.95 eV, respectively (see Figure 3). The valence band top (VBT) in both of the spin-up and spin-down band structures have dispersion features, indicating the improvement of carrier mobility with the functionalization. However, the conduction band bottom (CBB) of the spin-up states of g-C_3_N_4_-zz4-W is almost flat, while that of the spin-down states keeps dispersion, revealing the coupling between the localized spin-polarization and carrier. The analysis of partial density of states (PDOS) further reveals the mechanism of ferromagnetism induced by the coupling. From the calculated PDOSs of W, N, and C (Figure 4), we can see that the conduction bottom states in both spin-up and spin-down bands are mainly attributed to the *d *electrons of W (Figure 4a), although the *p *electrons from C and N (Figure 5b, c, d, e, f) also partially contribute to these states. Interestingly, the *s *electrons of W only contribute to the spin-up CBB states (Figure 4a). The VBT states are mainly attributed to the *d *electrons of W and the *p *electrons of N (Figure 4a, d, e, f). The unsymmetrical features of the PDOSs reveal that W-d, W-s, C-p, and N-p electrons are spin-polarized in the spin-up states. The coupling among the spin-polarized W-*d*, W-*s*, C-*p *and N-*p *electrons results in the alignment of magnetic moments and thus the ferromagnetism of W-functionalized graphitic carbon nitride nanotubes. The magnetic moment is about 3.82 μ_B _per W (see Table 1). The calculated band structures and PDOSs suggest that the ferromagnetism in the W-functionalized nanotube can be explained by the carrier-mediated interaction because the impurity bands are close to the CBBs, which is one of important mechanisms for the ferromagnetism in dilute magnetic semiconductors [1,3,14-16,35]. The carriers, i.e., electrons, introduced by the functionalization of W, mediate the alignment of the magnetic moments via the hybridization and coupling of spin-polarized *p *and *d *electrons (Figure 4).

**Figure 6:**
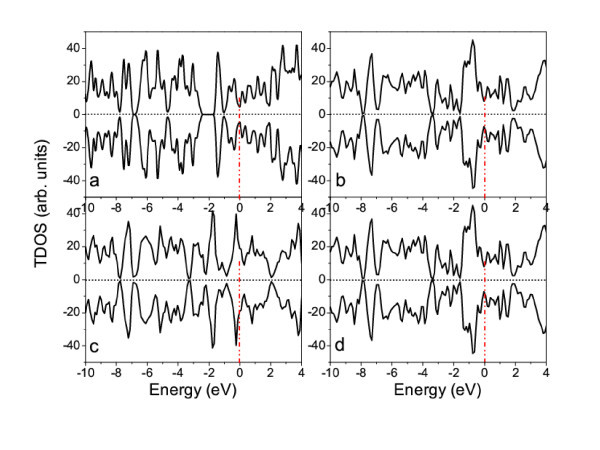
**Calculated TDOSs of graphitic carbon nitride nanotubes functionalized by metals**. **(a)** Cr, **(b) **Mn, **(c) **Co, and (d) Ni. The Fermi level is shifted to 0 eV (dot-dash line).

**Figure 7:**
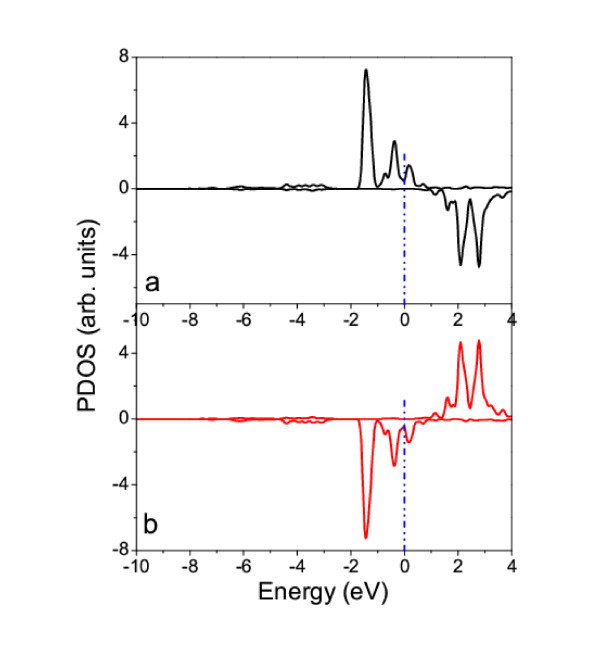
**Calculated PDOSs of two neighboring Cr atoms in Cr-functionalized g-C_3_N_4_-zz4**. The Fermi level is shifted to 0 eV (dot-dash line).

Figure 5 shows the calculated electronic structures of Ti-functionalized nanotube. The g-C_3_N_4_-zz4-Ti is also a heavily-doped n-type semiconductor with the direct gaps of approximately 1.4 and 1.6 eV in spin-up and spin-down band structures (Figure 5a,b). Similar to those of W-functionalized nanotube, the dispersion features in the both of the spin-up and spin-down band structures show the improvement of carrier mobility due to the functionalization. The CBB of the spin-up and spin-down states of g-C_3_N_4_-zz4-Ti are also dispersion, except flat levels appear above the bottoms, indicating the coupling between the localized spin-polarization and carrier, which leads to the ferromagnetism of graphitic carbon nitride nanotubes. The ferromagnetism of the Ti-functionalized nanotube is further confirmed by the unsymmetrical TDOS (Figure 5c). The spin-polarized impurity states within the CBB are mainly attributed to the Ti-*d *electrons (see Figure 5d). Similar to g-C_3_N_4_-zz4-W, the coupling among the spin-polarized Ti-*d*, C-*p*, and N-*p *electrons results in the alignment of the magnetic moments, and thus the ferromagnetism of Ti-functionalized graphitic carbon nitride nanotube (Figure 5d). The magnetic moment is about 2.88 μ_B _per Ti atom (Table 1). The ferromagnetism of Ti-functionalized nanotube is also attributed to carrier-mediated interaction because the impurity bands are close to the band edges and the Fermi level is within the conduction bands [15,35],

In contrast to W- and Ti-functionalized nanotubes, the Cr-, Mn-, Co-, and Ni-functionalized nanotubes are anti-ferromagnetic, as indicated by the calculated electronic structures (Figure 6). The PDOS analysis reveals that the *d *electrons of TM atoms (Cr, Mn, Co, and Ni) are spin-polarized, but the magnetic moments belonging to two neighboring atoms are anti-parallel, resulting in the anti-ferromagnetism (Figure 7).

## Conclusions

In summary, we studied the magnetic properties of metal-functionalized graphitic carbon nitride nanotubes based on the first-principles calculations. The results show that magnetic properties strongly depend on the coupling between the *d *electrons of metal atoms and *p *electrons of C or N atoms. The coupling and hybridization between the spin-polarized *d *electrons of W or Ti atoms and *p *electrons of C and N atoms result in the ferromagnetic W- and Ti-functionalized nanotubes. Although the d electrons of Cr, Mn, Co, and Ni atoms are spin-polarized, the functionalized nanotubes are anti-ferromagnetic because the spin-polarized *d *electrons are anti-parallel due to lack of coupling. The ferromagnetic nanotubes may be used in spintronics and other magnetic devices. The functionalized nanotubes may also find potential applications, such as hydrogen and energy storage [36-38].

## Abbreviations

CBB: conduction band bottom; DMSs: dilute magnetic semiconductors; g-C_3_N_4_: graphitic carbon nitride; PAW: projector augmented wave; PDOS: partial density of states; TM: transition metal; VASP: Vienna ab initio simulation package; VBT: valence band top.

## Competing interests

The authors declare that they have no competing interests.

## Authors' contributions

HP designed the ideas, performed the calculations and analysis of the results, and drafted the manuscript. YWZ, VBS, and HJG did the analysis of the results.
